# RUNX1-ETO (RUNX1-RUNX1T1) induces myeloid leukemia in mice in an age-dependent manner

**DOI:** 10.1038/s41375-021-01268-4

**Published:** 2021-06-19

**Authors:** Mohamed Gaber Abdallah, Akiko Niibori-Nambu, Mariko Morii, Takako Yokomizo, Tomomasa Yokomizo, Takako Ideue, Sho Kubota, Vania Swee Imm Teoh, Michelle Meng Huang Mok, Chelsia Qiuxia Wang, Abdellah Ali Omar, Kenji Tokunaga, Eisaku Iwanaga, Masao Matsuoka, Norio Asou, Naomi Nakagata, Kimi Araki, Mabrouk AboElenin, Sayed Hamada Madboly, Goro Sashida, Motomi Osato

**Affiliations:** 1 International Research Center for Medical Sciences, Kumamoto University, Kumamoto, Japan; 2 Department of Medical Biochemistry, Faculty of Medicine, Al-Azhar University, Cairo, Egypt; 3 Cancer Science Institute of Singapore, National University of Singapore, Singapore, Singapore; 4 Department of Tumor Genetics and Biology, Graduate School of Medical Sciences, Institute of Life Sciences, Kumamoto University, Kumamoto, Japan; 5 Department of Hematology, Rheumatology, and Infectious Diseases, Kumamoto University School of Medicine, Kumamoto, Japan; 6 Department of Hematology, International Medical Center, Saitama Medical University, Saitama, Japan; 7 Division of Reproductive Engineering, Center for Animal Resources and Development, Kumamoto University, Kumamoto, Japan; 8 Institute of Bioengineering and Nanotechnology, A*STAR, Singapore, Singapore; 9 Department of Paediatrics, National University of Singapore, Singapore, Singapore

**Keywords:** Cancer genetics, Acute myeloid leukaemia

## To the Editor:

t(8;21) is among the most common chromosomal translocations associated with human leukemia [1–4]. The *RUNX1-ETO (RUNX1-RUNX1T1, AML1-MTG8)* fusion gene, generated by t(8;21), has been extensively investigated in the field; however, its mechanistic basis remains to be fully understood. Clinical features of t(8;21) leukemia include: (1) association with acute myeloid leukemia (AML) M2 subtype in the FAB classification, characterized by granulocytic maturation in morphology, (2) positivity for the following immunophenotypic markers, HLA-DR^+^, CD117(c-KIT)^+^, CD34^+^, CD38^+^, CD13^+^, CD33^+^, CD19^+^, and CD56^+^, (3) chloroma, (4) predominant onset in adolescent and young adults (AYA), but not in aged population, (5) higher prevalence in Asia, and (6) requirement of additional genetic abnormalities such as mutations in *c-KIT, FLT3, RAS, ASXL1*, and *ZBTB7A*, -9q, or –Y [2, 3]. Although a number of attempts have been made to generate its mouse model, RUNX1-ETO induction alone did not induce leukemia in most cases [5–10]. Even when leukemia developed, the penetrance was low and the latency was 1 year or longer. In addition, leukemia phenotypes did not recapitulate clinical features. As such, no tractable murine models are currently available.

**Fig. 1:**
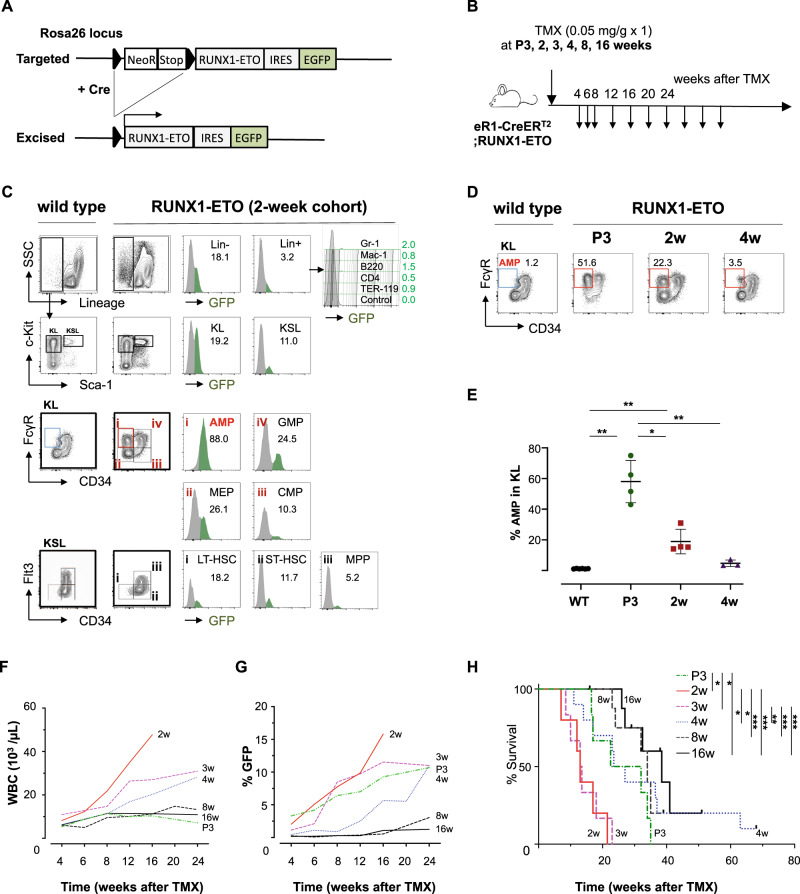
RUNX1-ETO induces abnormal myeloid progenitor (AMP) at pre-leukemic stage. **A** Schematic representation for the Rosa26-LSL(LoxP-Stop-LoxP)-RUNX1-ETO-IRES-EGFP targeted and LSL excised alleles. LoxP sequences are indicated as black triangles. In the presence of Cre recombinase, the LSL cassette is excised and the inserted RUNX1-ETO and EGFP genes are induced by ubiquitously active Rosa26 promoter. GFP serves as a surrogate marker for RUNX1-ETO expression. **B** Experimental design for tamoxifen (TMX) injection and follow-up intervals of serial collection of peripheral blood (PB). TMX (0.05 mg/g ×1) was intraperitoneally injected at indicated ages. Collected PB is subjected to complete blood cell counts (CBC) and flow cytometry analysis for frequency check of GFP^+^ cells. **C** Representative flow cytometry profiles at asymptomatic pre-leukemia status in the RUNX1-ETO mice showing the expansion of abnormal myeloid progenitor (AMP). Bone marrow (BM) cells collected from the RUNX1-ETO and littermate wild-type control mice in 2-week cohort at 4 weeks after TMX injection were analyzed. Contour plots at left-end two columns represent frequencies of indicated compartments of hematopoietic stem progenitor cells (HSPCs). Histograms at middle two columns exhibit frequencies of GFP^+^ cells in the corresponding fractions gated in the contour plots. Numbers in the histograms represent GFP percentages. (**D**, **E**) An age-dependent difference in the frequencies of AMP. Representative flow profiles for AMP at three distinct age cohorts and control (**D**) and mean frequencies of AMP in c-Kit^+^Sca-1^−^Lin^−^ (KL) populations (WT, P3, 2w, *n* = 4; 4w, *n* = 3) (**E**) are shown. Asterisk(s) represents significant differences [*P ≤ 0.05, **P ≤ 0.01, ***P ≤ 0.001, two-way analysis of variance (ANOVA) with subsequent Bonferroni test]. Time course kinetics of white blood cell counts (WBC) (**F**) and percentages of GFP^+^ cells (**G**) in the PB. Individual lines represent mean values of parameters in individual cohorts, excluding diseased mice at final stages. **H** Kaplan–Meier survival curves of RUNX1-ETO mice in individual cohorts. 2- and 3-week cohorts show significantly shorter survival as compared to other indicated cohorts (P3, *n* = 6; 2w, *n* = 5; 3w, *n* = 6; 4w, *n* = 10; 8w, *n* = 8; 16w, *n* = 8). Asterisk(s) represents significant differences (*P ≤ 0.05, **P ≤ 0.01, ***P ≤ 0.001, Log-rank test). Abbreviations: KL, c-Kit^+^Sca-1^-^Lin^-^; KSL, c-Kit^+^Sca-1^+^Lin^−^; LT-HSC, long-term hematopoietic stem cells; ST-HSC short term HSCs, CMP common myeloid progenitors, GMP granulocyte macrophage progenitors, MEP megakaryocyte erythrocyte progenitors.

We reasoned that the failure in the previous efforts for the generation of RUNX1-ETO mouse model is due to insufficient expression and inappropriate cells of origin. RUNX1-ETO mRNA amount per one single leukemia cell at clinical onset time is shown to be significantly higher than that at remission state [11]. To achieve sufficient expression, Rosa26 locus which allows for abundant expression of RUNX1-ETO was employed to generate conditional knock-in (KI) mice carrying a heterozygous floxed allele of Rosa26-LSL-RUNX1-ETO-IRES-EGFP (Fig. 1A, Tables S1 and S2, Supplementary Methods). This mouse line was crossed with eR1-CreER^T2^ transgenic mice (Tg) which targets RUNX1-ETO expression predominantly to hematopoietic stem cells (HSCs) [12]. In all earlier studies by others, induction of RUNX1-ETO in the conditional models was conducted at adult stages. However, the *RUNX1-ETO* gene was documented to be detectable as early as in neonates [13]. Therefore, in this study, the RUNX1-ETO was induced at various ages including childhood [postnatal day 3 (P3), 2-, 3- and 4-week-old] besides adult (8- and 16-week-old) (Fig. 1B). eR1-CreER^T2^ Tg; Rosa26-LSL-RUNX1-ETO-IRES-EGFP mice are referred to as RUNX1-ETO mice, and individual induction groups are stated to be P3 or 2-, 3-, 4-, 8- and 16-week cohorts, respectively. For complete information about the generation of mice, hematological and flow cytometric analyses, and other experimental details, see Supplemental Methods.

The efficiency of RUNX1-ETO induction by a single injection of tamoxifen (TMX, 0.05 mg/g) was first examined by flow cytometry. As GFP acts as a surrogate marker of RUNX1-ETO induction, the comparison of frequencies of GFP^+^ cells at 24 h after TMX injection confirmed comparable induction efficiencies between distinct age groups (P3 versus 4-week cohorts, Fig. S1A, B, Table S3). Next, to identify the initial effects of the RUNX1-ETO induction on hematopoietic cells, hematological analyses were conducted 1 month after TMX injection. Flow cytometry analysis exhibited prominent expansion of c-Kit^+^Sca-1^‒^Lin^‒^CD34^‒^FcγR^+^ compartment, hereinafter referred to as abnormal myeloid progenitor (AMP), at various frequencies in childhood cohorts (Figs. 1C–E and S1C–F). The frequency of AMP declined with age and was already very low in the 4-week cohort. Kuo et al. reported that an immunophenotypically-similar AMP fraction is also generated in another RUNX1 related mouse model carrying Cbfβ-SMMHC fusion gene which inhibits RUNX1 function like RUNX1-ETO [14]. The formation of AMPs in two distinct mouse models is a good indication that our RUNX1-ETO model is a suitable platform to address the core molecular effects directly induced by the fusion gene at the pre-leukemic stage.

White blood cell counts and GFP^+^ percentages in the peripheral blood serially collected from the TMX-administered mice exhibited a gradual increase of RUNX1-ETO-expressing cells in an age-dependent manner (Figs. 1F, G and S1G). Starting from 8 weeks after TMX induction, RUNX1-ETO mice died due to hematological malignancies (Fig. 1H), characterized by leukocytosis, anemia, thrombocytopenia, and hepatosplenomegaly. Two- and three-week cohort mice, followed by P3 cohort, developed diseases with short latency and complete penetrance, whereas 4-, 8- and 16-week cohort mice exhibited a long latency and/or incomplete penetrance.

**Fig. 2:**
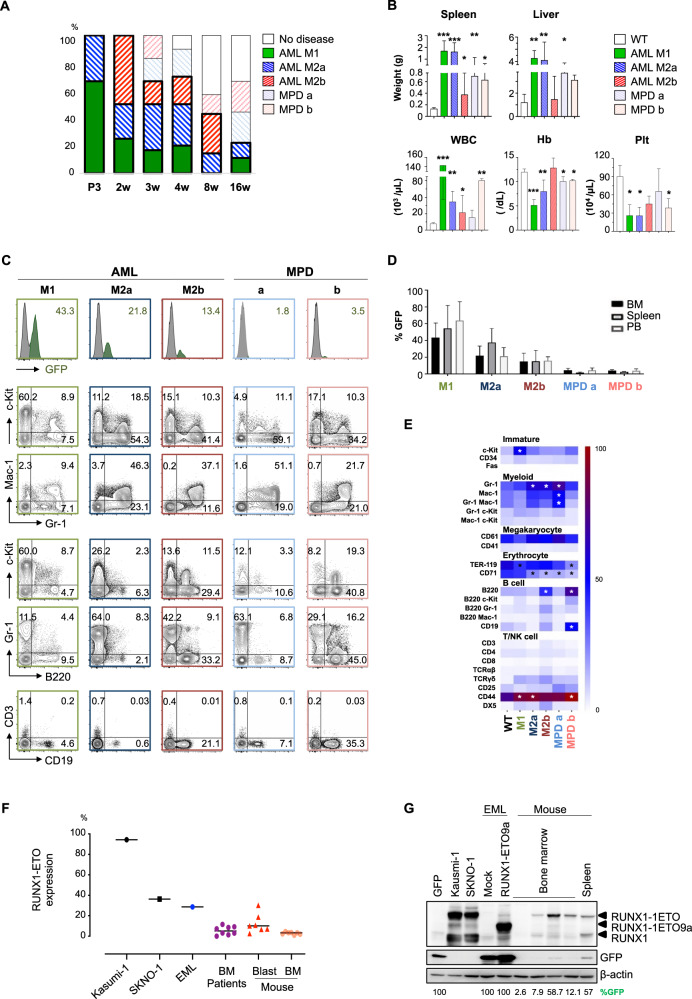
RUNX1-ETO induces an age-dependent myeloid spectrum disorder. **A** Incidence of indicated acute myeloid leukemia (AML) and myeloproliferative disorder (MPD) subtypes in each age cohort. **B** Weight of spleen and liver, white blood cells (WBC), hemoglobin (Hb), and platelet (Plt) counts of mice of indicated disease subtypes and wild type (WT). Asterisk(s) represents significant differences (*P ≤ 0.05, **P ≤ 0.01, ***P ≤ 0.001, Student t-test). **C** Representative flow cytometry profiles of malignant cells from indicated disease subtypes. Histograms at top row show GFP% in the BM. Contour plots below exhibit positivity for indicated antigens on total bone marrow (BM) cells. **D** Frequency of GFP^+^ cells in the BM, Spleen and peripheral blood (PB) in indicated disease subtypes. **E** Heatmap for the percentages of individual antigen^+^ cells in total BM cells (M1, *n* = 7; M2a, *n* = 9; M2b, *n* = 7; MPD a, *n* = 5; MPD b, *n* = 3). Asterisk(s) in white and black represents a significant increase or decrease, respectively, against the corresponding WT population [*P ≤ 0.05, two-way ANOVA with subsequent Bonferroni test]. **F**, **G** Expression levels of RUNX1-ETO in the present mouse model, human clinical cases, and commonly used experimental materials such as human leukemia cell lines (Kasumi-1 and SKNO-1) and a mouse leukemia cell line EML retrovirally traduced with RUNX1-ETO9a, in quantitative reverse transcription-polymerase chain reaction (**F**) and western blot analysis (**G**). **F** mRNA expression level of RUNX1-ETO is shown as % against that in Kasumi-1. BM patients, blast, or BM from mouse represent non-sorted mononuclear cells from t(8;21) leukemia patients (*n* = 8), sorted GFP^+^c-Kit^+^Gr1^−^B220^−^ blast cells (*n* = 7), or non-sorted whole bone marrow cells (n = 7) from this mouse model, respectively. **G** Protein expression levels of RUNX1-ETO, GFP, and β-actin (an internal control) in the indicated materials are shown. %GFP in individual samples are given at the bottom of panels.

Hematological malignancies were classified into five distinct subtypes, according to their GFP percentages, immunophenotypic and morphological features (Table S4, Figs. 2, S2A–D, and S3A). Diseases with 10% or above GFP^+^ cells in any single hematopoietic tissues were diagnosed as leukemia, based on the result of Euclidian distance analysis as stated in the footnote for Table S4. In addition, according to their differentiation property, leukemia was further classified into three subtypes: AML M1, characterized by c-Kit^high^ immature myeloid cells without maturation; AML M2a, with granulocytic maturation (Gr-1 ≥ 30%, B220 < 30%); and AML M2b, exhibiting granulocytic maturation with B cell features (Gr-1 ≥ 30%, B220 ≥ 30%). In the morphological analysis, immature myeloblasts with basophilic cytoplasm were prevalent in M1 subtype, whereas mature myeloid cells were expanded in M2 subtypes (Fig. S3A). Surprisingly, some diseased mice did not show any obvious increase in GFP^+^ cells (Fig. 2C, D), although they definitely suffered from fatal symptoms including huge splenomegaly (Fig. 2B). As their blood cells revealed granulocytic maturation, the disease was considered myeloproliferative disorder (MPD). Based on their features of differentiation, MPD cases were classified into two subtypes, MPD a (Gr-1 ≥ 30%, B220 < 30%) and MPD b (Gr-1 ≥ 30%, B220 ≥ 30%). To exclude the possibility of silencing of GFP expression in the MPD cases, namely the residual RUNX1-ETO expression in the GFP^-^ fraction, semi-quantitative polymerase chain reaction (PCR) and quantitative reverse transcription-PCR (qRT-PCR) were conducted on genomic DNAs and RNAs extracted from 6 MPD cases, respectively (Fig. S3B, C). The results showed a good correlation of GFP^+^ percentages with the intensities of excised bands and the RUNX1-ETO mRNA expression level. Cell differentiation-related silencing was also not observed, as abundant expression of RUNX1-ETO mRNA was confirmed in sorted Gr-1^+^ cells (Fig. S3D). Furthermore, transplantation assay clearly demonstrated no disease development in the recipients of sorted GFP^‒^ cells from MPD cases, although the recipients of GFP^+^ cells or whole bone marrow cells developed leukemia (Fig. S4). Leukemia subtypes in these recipients were more aggressive than original subtypes seen in the primary diseased mice. Therefore, the GFP silencing phenomenon was unlikely and we postulate that the small number of RUNX1-ETO expressing cells may cause hematological malignancies in a non-cell autonomous manner in the MPD cases (Fig. S3F). Besides differences in immunophenotypic markers, AMP frequencies and disease latencies also varied amongst disease subtypes (Figs. S2E–G and S3E). To exclude leakiness of the inducible system, 4 non-TMX-administered mice were observed for at least 10 months, and no GFP^+^ cells were detected during this period (data not shown).

Besides latency and penetrance, the incidence of disease subtypes also differed with age of RUNX1-ETO induction (Fig. 2A). AML M1 was predominantly seen in P3 cohort and declined with age. M2a was persistent throughout all cohorts, whereas M2b subtype was only seen from 2- to 8-week cohorts. MPD occurred in 3-week or later cohorts. Such age-dependent development of malignant myeloid diseases, particularly M2a and M2b subtypes, seems to recapitulate clinical features seen in human t(8;21) leukemia, such as AYA onset and AML M2 subtype characterized by granulocytic maturation with B cell features. MPD subtypes in this model also mimic human t(8;21) disease, as t(8;21) is found in smoldering myelodysplastic syndrome as well [15]. Extramedullary manifestation, such as chloroma in the skin, is well documented to occur in t(8;21) leukemia patients [4]. Interestingly, skin lesions in the face (eyelid, mouth, and ear), limbs, and tail were also observed in this mouse model in an age- and TMX dosage-dependent manner. As NRAS mutations are frequently found in human t(8;21) leukemias, Nras G12D mutation was additionally introduced into the RUNX1-ETO model. When TMX was injected at 4 weeks old, eR1-CreER^T2^ Tg; Rosa26-LSL-RUNX1-ETO-IRES-EGFP; Rosa26-LSL-NrasG12D mice developed leukemia with complete penetrance and shorter latency as compared to RUNX1-ETO alone model (Fig. S5). This result suggests that Nras G12D accelerates leukemia development by RUNX1-ETO, and RUNX1-ETO per se might be insufficient for full-blown leukemogenesis. Lastly, qRT-PCR and western blot analyses demonstrated that the expression level of RUNX1-ETO mRNA and protein in this mouse model is comparable to that in t(8;21) clinical samples, and not as high as those in RUNX-ETO expressing human leukemia cell lines, Kasumi-1 and SKNO-1, and a mouse cell line retrovirally transduced with RUNX-ETO9a (Fig. 2F, G). Altogether, our newly generated RUNX1-ETO model appears to be a long-awaited clinically relevant tractable t(8;21) leukemia murine model that will serve as a platform for molecular dissection of leukemogenesis and drug efficacy testing. The non-cell autonomous and age-dependent t(8;21) leukemogenesis unveiled and confirmed in this model will provide us with novel insights into the mechanistic basis and novel therapeutic strategy.

## Supplementary information

Suplementary methods

Suplementary information

## References

[1] Metzeler KH, Bloomfield CD (2017). Clinical relevance of RUNX1 and CBFB alterations in acute myeloid leukemia and other hematological disorders. Adv Exp Med Biol.

[2] Bolouri H, Farrar JE, Triche T, Ries RE, Lim EL, Alonzo TA (2018). The molecular landscape of pediatric acute myeloid leukemia reveals recurrent structural alterations and age-specific mutational interactions. Nat Med.

[3] Jiang L, Li XP, Dai YT, Chen B, Weng XQ, Xiong SM (2020). Multidimensional study of the heterogeneity of leukemia cells in t(8;21) acute myelogenous leukemia identifies the subtype with poor outcome. Proc Natl Acad Sci USA.

[4] Reikvam H, Hatfield KJ, Kittang AO, Hovland R, Bruserud Ø (2011). Acute myeloid leukemia with the t (8; 21) translocation: clinical consequences and biological implications. J Biomed Biotechnol.

[5] Chin D, Watanabe-Okochi N, Wang C, Tergaonkar V, Osato M (2015). Mouse models for core binding factor leukemia. Leukemia..

[6] Rhoades KL, Hetherington CJ, Harakawa N, Yergeau DA, Zhou L, Liu LQ (2000). Analysis of the role of AML1-ETO in leukemogenesis, using an inducible transgenic mouse model. Blood..

[7] Yuan Y, Zhou L, Miyamoto T, Iwasaki H, Harakawa N, Hetherington CJ (2001). AML1-ETO expression is directly involved in the development of acute myeloid leukemia in the presence of additional mutations. Proc Natl Acad Sci USA.

[8] Higuchi M, O’Brien D, Kumaravelu P, Lenny N, Yeoh E-J, Downing JR (2002). Expression of a conditional AML1-ETO oncogene bypasses embryonic lethality and establishes a murine model of human t (8; 21) acute myeloid leukemia. Cancer Cell.

[9] Fenske TS, Pengue G, Mathews V, Hanson PT, Hamm SE, Riaz N (2004). Stem cell expression of the AML1/ETO fusion protein induces a myeloproliferative disorder in mice. Proc Natl Acad Sci USA.

[10] Cabezas-Wallscheid N, Eichwald V, de Graaf J, Lower M, Lehr HA, Kreft A (2013). Instruction of haematopoietic lineage choices, evolution of transcriptional landscapes and cancer stem cell hierarchies derived from an AML1-ETO mouse model. EMBO Mol Med.

[11] Shima T, Miyamoto T, Kikushige Y, Yuda J, Tochigi T, Yoshimoto G (2014). The ordered acquisition of Class II and Class I mutations directs formation of human t(8;21) acute myelogenous leukemia stem cell. Exp Hematol.

[12] Ng CEL, Yokomizo T, Yamashita N, Cirovic B, Jin H, Wen Z (2010). A Runx1 intronic enhancer marks hemogenic endothelial cells and hematopoietic stem cells. Stem Cells.

[13] Wiemels JL, Xiao Z, Buffler PA, Maia AT, Ma X, Dicks BM (2002). In utero origin of t (8; 21) AML1-ETO translocations in childhood acute myeloid leukemia. Blood..

[14] Kuo Y-H, Landrette SF, Heilman SA, Perrat PN, Garrett L, Liu PP (2006). Cbfβ-SMMHC induces distinct abnormal myeloid progenitors able to develop acute myeloid leukemia. Cancer Cell.

[15] Yamasaki H, Era T, Asou N, Sanada I, Matutes E, Yamaguchi K (1995). High degree of myeloid differentiation and granulocytosis is associated with t(8;21) smoldering leukemia. Leukemia..

