# Supplementary material for: RUNX1-ETO (RUNX1-RUNX1T1) induces myeloid leukemia in mice in an age-dependent manner
Source: Leukemia. 2021 Jun 19;35(10):2983–8. doi: 10.1038/s41375-021-01268-4 (PMC8478654; doi:10.1038/s41375-021-01268-4)
Supplement: Supplementary file 1 — Suplementary methods [file 41375_2021_1268_MOESM1_ESM.pptx]

## Slide 1
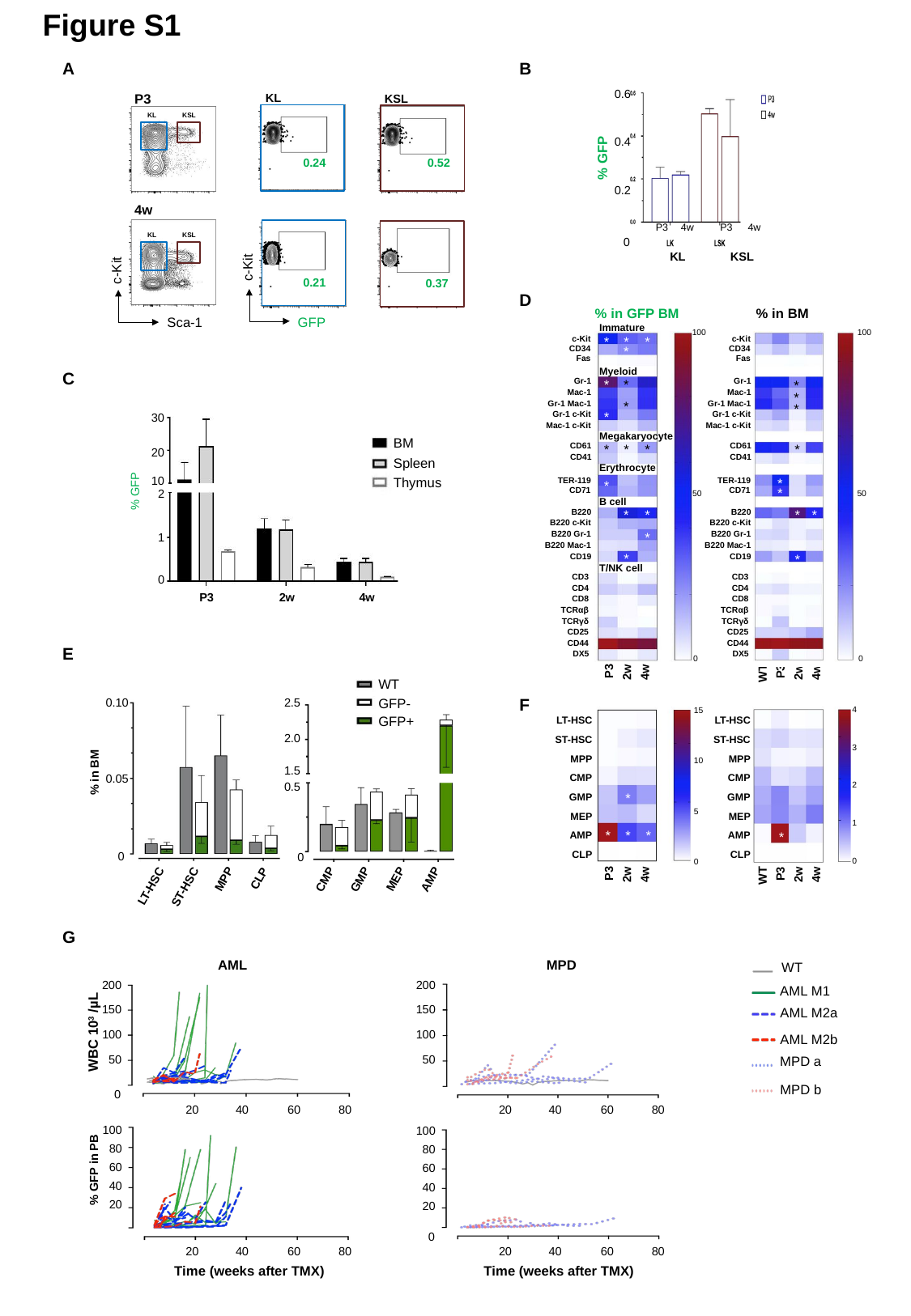

Figure S1
A
B
0.6
0.4
% GFP
0.2
P3
4w
P3
4w
0
KL
KSL
P3
KL
KSL
KL
KSL
0.24
0.52
4w
c-Kit
Sca-1
KL
KSL
c-Kit
GFP
0.21
0.37
D
% in BM
% in GFP BM
Immature
100
100
*
*
*
c-Kit
CD34
Fas
Gr-1
Mac-1
Gr-1 Mac-1
Gr-1 c-Kit
Mac-1 c-Kit
CD61
CD41
TER-119
CD71
B220
B220 c-Kit
B220 Gr-1
B220 Mac-1
CD19
CD3
CD4
CD8
TCRαβ
TCRγδ
CD25
CD44
DX5
c-Kit
CD34
Fas
Gr-1
Mac-1
Gr-1 Mac-1
Gr-1 c-Kit
Mac-1 c-Kit
CD61
CD41
TER-119
CD71
B220
B220 c-Kit
B220 Gr-1
B220 Mac-1
CD19
CD3
CD4
CD8
TCRαβ
TCRγδ
CD25
CD44
DX5
*
Myeloid
*
*
*
*
*
*
*
Megakaryocyte
*
*
*
*
Erythrocyte
*
*
*
50
50
B cell
*
*
*
*
*
*
*
T/NK cell
*
0
0
P3
2w
4w
C
30
BM
Spleen
Thymus
20
10
2
% GFP
1
0
P3
2w
4w
E
P3
2w
4w
WT
WT
GFP-
GFP+
0.10
2.5
2.0
% in BM
1.5
0.05
0.5
0
0
CMP
GMP
MEP
AMP
LT-HSC
ST-HSC
MPP
CLP
F
4
15
LT-HSC
ST-HSC
MPP
CMP
GMP
MEP
AMP
CLP
LT-HSC
ST-HSC
MPP
CMP
GMP
MEP
AMP
CLP
3
10
2
*
5
1
*
*
*
*
0
0
P3
2w
4w
P3
2w
4w
WT
G
AML
MPD
WT
AML M1
AML M2a
AML M2b
MPD a
MPD b
200
150
100
50
200
150
100
50
 WBC 103 /µL
0
20
40
60
80
20
40
60
80
100
80
60
40
20
100
80
60
40
20
% GFP in PB
0
20
40
60
80
20
40
60
80
Time (weeks after TMX)
Time (weeks after TMX)

## Slide 2
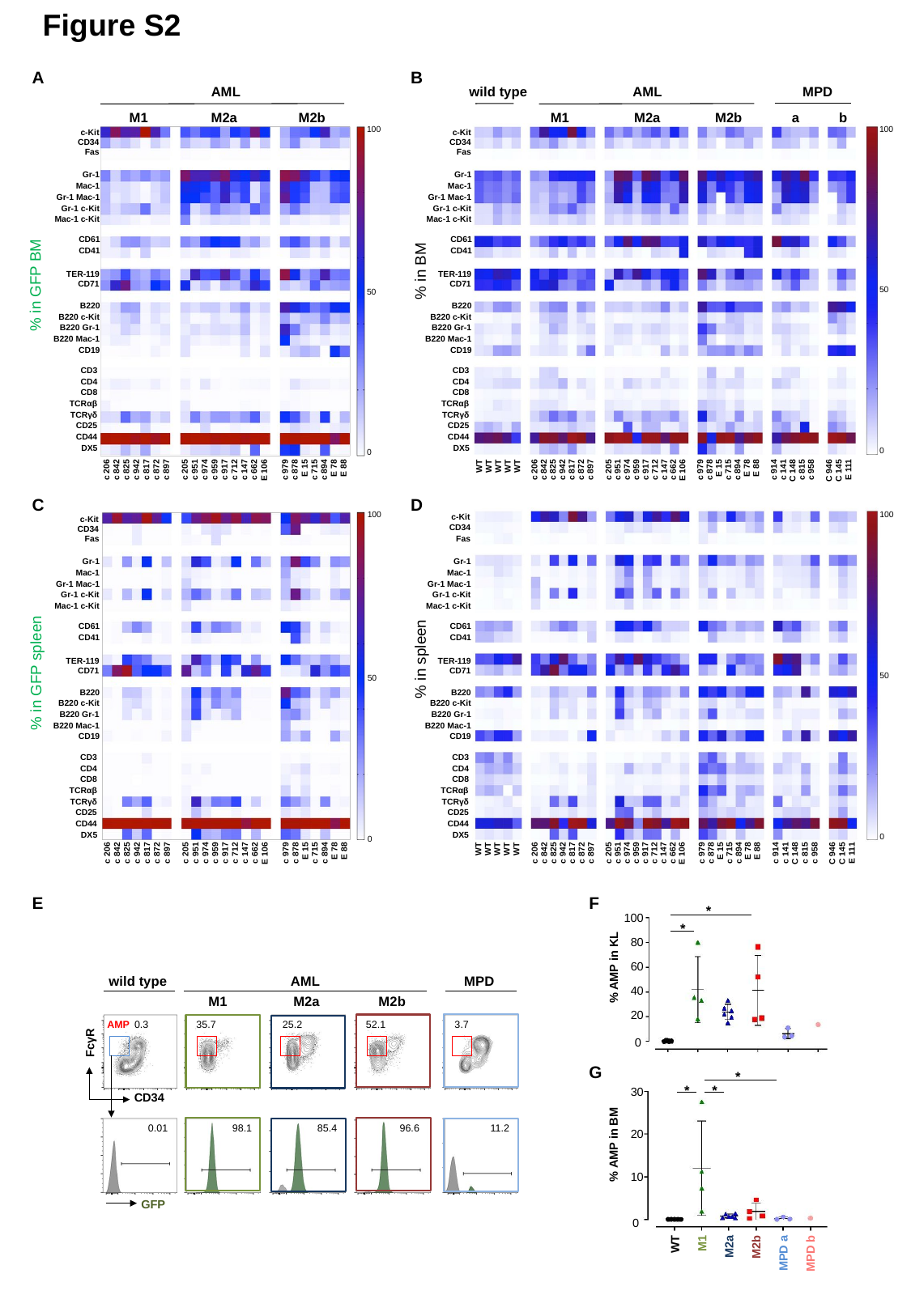

Figure S2
A
B
AML
M1
M2a
M2b
100
c-Kit
CD34
Fas
Gr-1
Mac-1
Gr-1 Mac-1
Gr-1 c-Kit
Mac-1 c-Kit
CD61
CD41
TER-119
CD71
B220
B220 c-Kit
B220 Gr-1
B220 Mac-1
CD19
CD3
CD4
CD8
TCRαβ
TCRγδ
CD25
CD44
DX5
% in GFP BM
50
0
c 206
c 842
c 825
c 942
c 817
c 872
c 897
c 205
c 951
c 974
c 959
c 917
c 712
c 147
c 662
E 106
c 979
c 878
E 15
c 715
c 894
E 78
E 88
wild type
AML
MPD
M1
M2a
M2b
a
b
100
c-Kit
CD34
Fas
Gr-1
Mac-1
Gr-1 Mac-1
Gr-1 c-Kit
Mac-1 c-Kit
CD61
CD41
TER-119
CD71
B220
B220 c-Kit
B220 Gr-1
B220 Mac-1
CD19
CD3
CD4
CD8
TCRαβ
TCRγδ
CD25
CD44
DX5
% in BM
50
0
WT
WT
WT
WT
WT
c 206
c 842
c 825
c 942
c 817
c 872
c 897
c 205
c 951
c 974
c 959
c 917
c 712
c 147
c 662
E 106
c 979
c 878
E 15
c 715
c 894
E 78
E 88
c 914
c 141
C 148
c 815
c 958
C 946
C 145
E 111
C
D
100
c-Kit
CD34
Fas
Gr-1
Mac-1
Gr-1 Mac-1
Gr-1 c-Kit
Mac-1 c-Kit
CD61
CD41
% in GFP spleen
TER-119
CD71
50
B220
B220 c-Kit
B220 Gr-1
B220 Mac-1
CD19
CD3
CD4
CD8
TCRαβ
TCRγδ
CD25
CD44
DX5
0
c 206
c 842
c 825
c 942
c 817
c 872
c 897
c 205
c 951
c 974
c 959
c 917
c 712
c 147
c 662
E 106
c 979
c 878
E 15
c 715
c 894
E 78
E 88
E
100
c-Kit
CD34
Fas
Gr-1
Mac-1
Gr-1 Mac-1
Gr-1 c-Kit
Mac-1 c-Kit
CD61
% in spleen
CD41
TER-119
CD71
50
B220
B220 c-Kit
B220 Gr-1
B220 Mac-1
CD19
CD3
CD4
CD8
TCRαβ
TCRγδ
CD25
CD44
DX5
0
WT
WT
WT
WT
WT
c 206
c 842
c 825
c 942
c 817
c 872
c 897
c 205
c 951
c 974
c 959
c 917
c 712
c 147
c 662
E 106
c 979
c 878
E 15
c 715
c 894
E 78
E 88
c 914
c 141
C 148
c 815
c 958
C 946
C 145
E 111
F
*
100
*
80
% AMP in KL
60
40
20
0
*
*
*
30
20
% AMP in BM
10
0
WT
M1
M2a
M2b
MPD a
MPD b
wild type
AML
MPD
M1
M2a
M2b
AMP
0.3
35.7
25.2
52.1
3.7
FcγR
G
CD34
0.01
98.1
85.4
96.6
11.2
GFP

## Slide 3
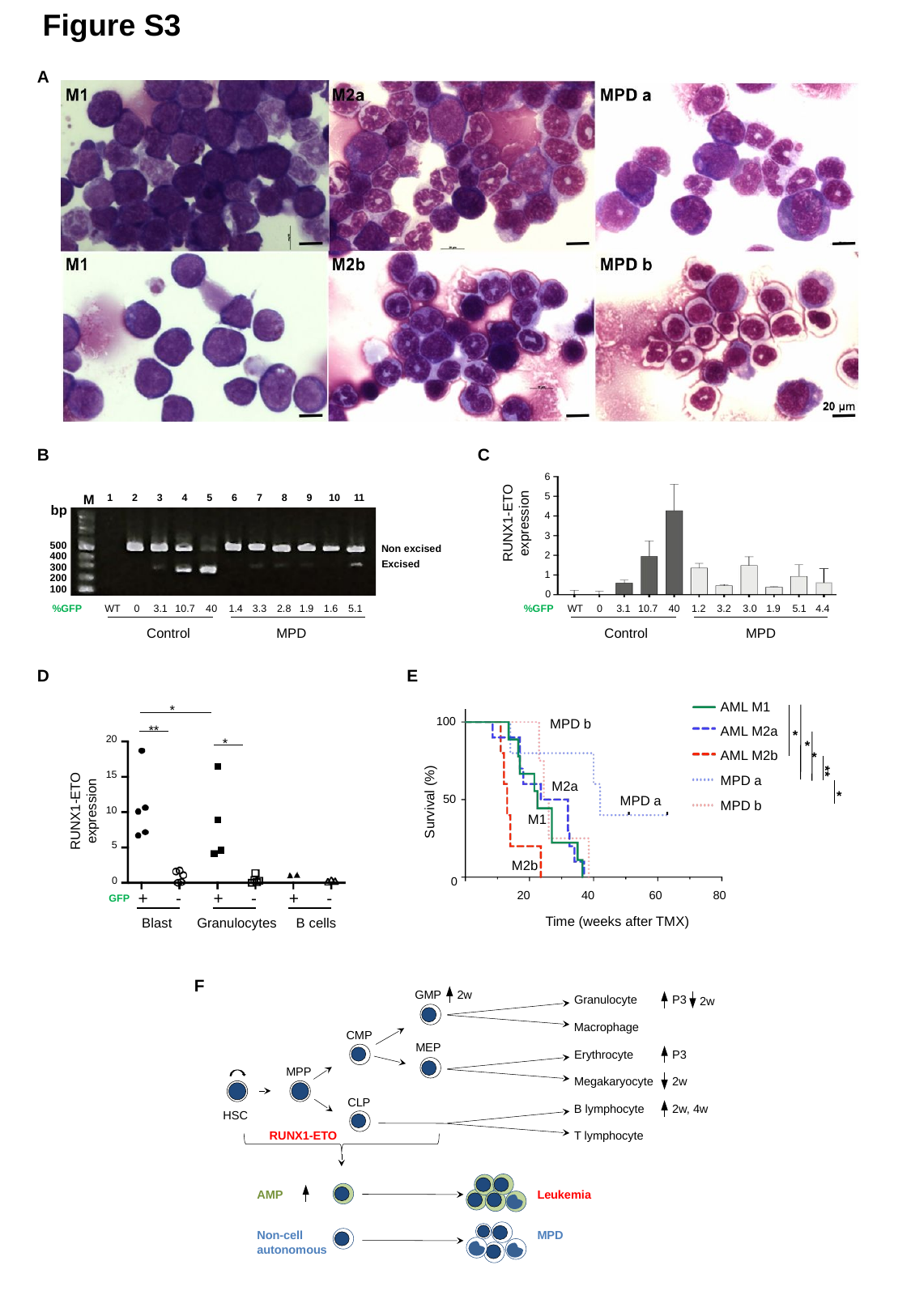

Figure S3
A
B
C
6
5
RUNX1-ETO expression
4
3
2
1
0
%GFP
WT
0
3.1
10.7
40
1.2
3.2
3.0
1.9
5.1
4.4
Control
MPD
M
1
2
3
4
5
6
7
8
9
10
11
bp
500
Non excised
400
Excised
300
200
100
%GFP
WT
0
3.1
10.7
40
1.4
3.3
2.8
1.9
1.6
5.1
Control
MPD
D
E
AML M1
AML M2a
AML M2b
MPD a
MPD b
*
*
*
**
*
*
**
20
*
15
RUNX1-ETO expression
10
5
0
+
-
+
-
+
-
GFP
Blast
Granulocytes
B cells
100
MPD b
 M2a
50
MPD a
Survival (%)
M1
 M2b
0
20
40
60
80
Time (weeks after TMX)
F
GMP
2w
Granulocyte
P3
 2w
Macrophage
CMP
MEP
Erythrocyte
P3
MPP
Megakaryocyte
2w
CLP
B lymphocyte
2w, 4w
HSC
RUNX1-ETO
T lymphocyte
AMP
Leukemia
Non-cell autonomous
MPD

## Slide 4
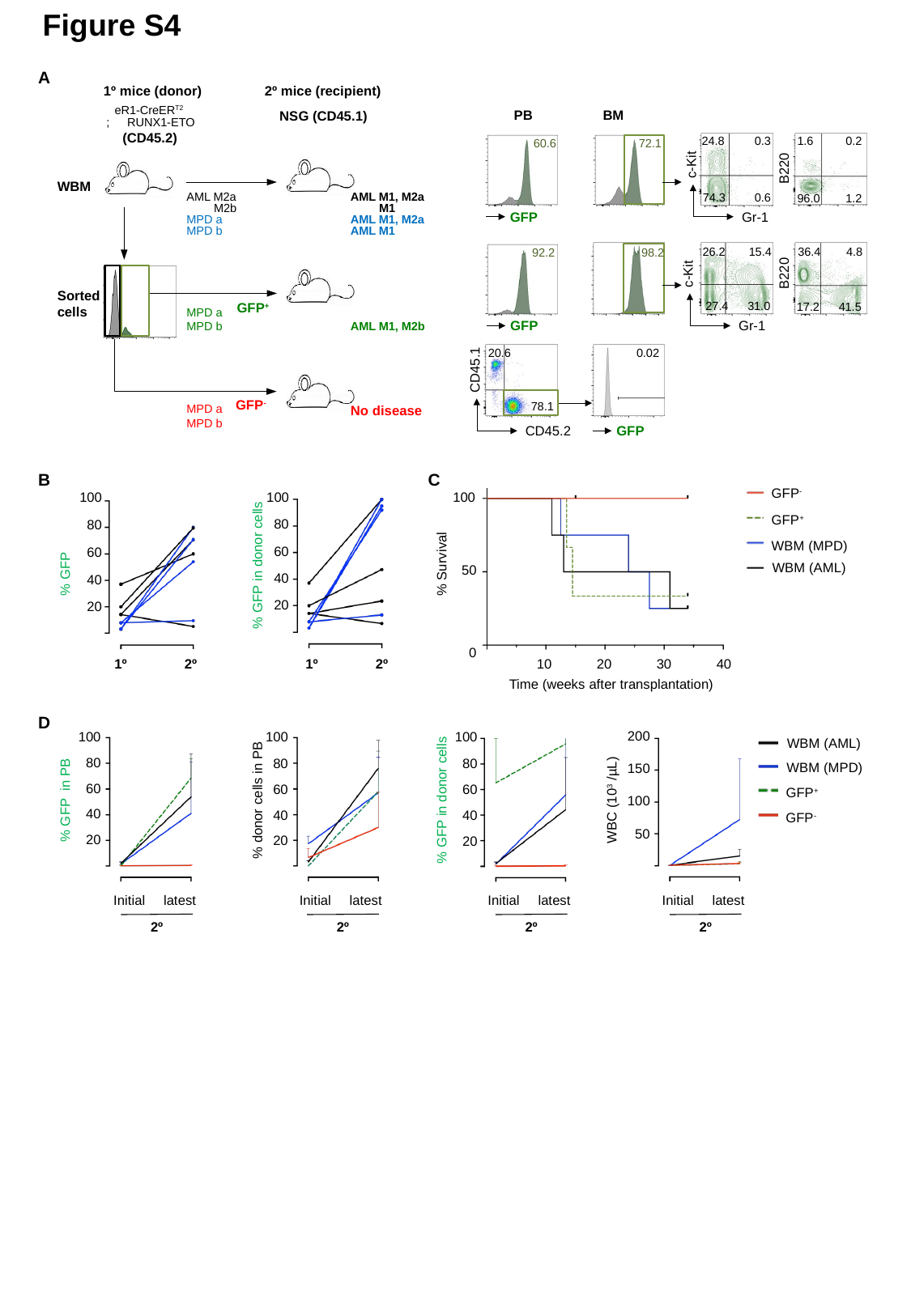

Figure S4
A
1º mice (donor)
2º mice (recipient)
eR1-CreERT2
;　RUNX1-ETO
PB
BM
c-Kit
Gr-1
24.8
0.3
1.6
0.2
60.6
72.1
B220
74.3
0.6
96.0
1.2
GFP
NSG (CD45.1)
(CD45.2)
WBM
AML M2a
AML M2b
MPD a
MPD b
AML M1, M2a
AML M1
AML M1, M2a
AML M1
c-Kit
Gr-1
26.2
15.4
36.4
4.8
98.2
92.2
B220
27.4
31.0
17.2
41.5
GFP
Sorted cells
GFP+
MPD a
MPD b
AML M1, M2b
CD45.1
CD45.2
20.6
0.02
78.1
GFP
GFP-
MPD a
MPD b
No disease
B
C
GFP-
100
100
100
GFP+
80
80
WBM (MPD)
60
60
% Survival
% GFP in donor cells
WBM (AML)
50
% GFP
40
40
20
20
0
1º
2º
1º
2º
10
20
30
40
Time (weeks after transplantation)
D
200
100
100
100
WBM (AML)
80
80
80
WBM (MPD)
150
60
60
60
GFP+
% GFP in PB
% donor cells in PB
% GFP in donor cells
WBC (103 /µL)
100
40
40
40
GFP-
50
20
20
20
Initial
latest
2º
Initial
latest
2º
Initial
latest
2º
Initial
latest
2º

## Slide 5
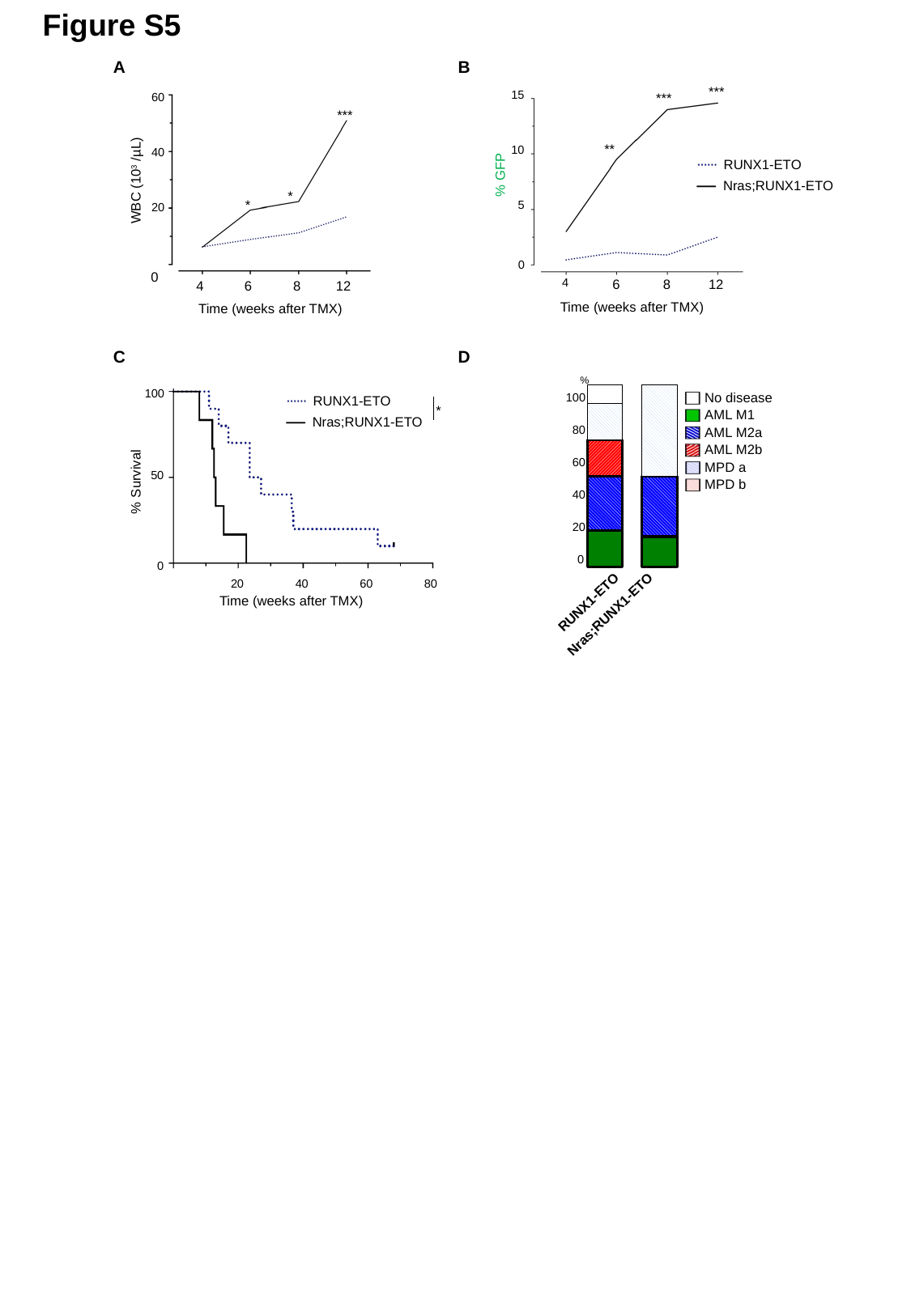

Figure S5
A
B
***
15
60
***
***
**
10
40
RUNX1-ETO
Nras;RUNX1-ETO
% GFP
WBC (103 /µL)
*
*
5
20
0
0
4
6
8
12
4
6
8
12
Time (weeks after TMX)
Time (weeks after TMX)
C
D
%
100
80
60
40
20
0
### Chart
| Category | AML M1 | AML M2a | AML M2b | MPD a | MPD b | No disease |
|---|---|---|---|---|---|---|
| 4w | 20.0 | 30.0 | 20.0 | 20.0 | 0.0 | 10.0 |
| 4w Nras | 16.6666666666667 | 33.3333333333333 | 0.0 | 50.0 | 0.0 | 0.0 |100
No disease
AML M1
AML M2a
AML M2b
MPD a
MPD b
RUNX1-ETO
*
Nras;RUNX1-ETO
50
% Survival
0
20
40
60
80
Time (weeks after TMX)
RUNX1-ETO
Nras;RUNX1-ETO
