# Supplementary material for: RUNX1-ETO (RUNX1-RUNX1T1) induces myeloid leukemia in mice in an age-dependent manner
Source: Leukemia. 2021 Jun 19;35(10):2983–8. doi: 10.1038/s41375-021-01268-4 (PMC8478654; doi:10.1038/s41375-021-01268-4)
Supplement: Supplementary file 2 — Suplementary information [file 41375_2021_1268_MOESM2_ESM.docx]

**SUPLEMENTARY METHODS**

**Mice**

To generate Rosa26-LSL-RUNX1-ETO-IRES-EGFP mice, a full length RUNX1-ETO, but not a truncated form RUNX1-ETO9a, was subcloned into the *Asc*I site of STOP-eGFP-ROSA26TV (Addgene cat#11739), a pBSII-KS based plasmid containing LoxP-Stop-LoxP (LSL) cassette for Cre recombinase-mediated deletion. For the combinational use of CRISPR/Cas (clustered regularly interspaced short palindromic repeats / CRISPR associated proteins) system, two sets of sgRNA oligos (listed in Table S1) were cloned into *Bbs*I-digested pX335-U6-Chimeric BB-CBh-hSpCas9n (D10A) plasmid (Addgene #42335).  C57BL/6N-derived ES cell line, 6NK7 ES cells (ref. 1) (2 x 10^6^ cells) were co-electroporated with circular forms of 20 μg of the targeting vector and 10 μg each of pX335-Rosa26-3 and pX335-Rosa26-4 using Gene Pulser Xcell^TM^ (Bio-Rad, Hercules, CA), and plated into two 10-cm plates. G418 selection was started after 24h of electroporation at 130 mg/ml for 7 days. On day 8, colonies were picked and stocked. Six distinct polymerase chain reaction (PCR)-positive ES clones were separately used to generate chimeric mice by aggregation with eight-cell embryos of ICR mice (Nippon Clea, Tokyo), at Center for Animal Resources and Development (CARD), Kumamoto University (KU), Japan. Of 6, two distinct chimeric mouse lines #15 and #4 were selected for further analyses. As these two lines did not exhibit any significant differences in the efficiency of LSL cassette excision and leukemogenesis, clone #15 derived knock-in (KI) mice were mainly used in the present study. Conditional KI mice carrying a floxed heterozygous allele of Rosa26-LSL-RUNX1-ETO-IRES-EGFP were crossed with previously generated eR1-CreER^T2^ transgenic (Tg) mice,^2^ to generate eR1CreER^T2^;Rosa26-LSL-RUNX1-ETO-IRES-EGFP offspring. Rosa26-LSL-RUNX1-ETO-IRES-EGFP mice were used as controls. For the activation of CreER^T2^ recombinase, mice received a single injection of intraperitoneal (IP) tamoxifen (TMX, Sigma T5648) at 0.05 mg/g at various ages as shown in Fig. 1B. TMX was dissolved in sunflower seed oil (Sigma S5007)/ethanol (10:1) mixture at 10 mg/ml. Nras^G12D^ (C57BL/6-*Nras^tm1Tyj^*/J) (ref. 3) and NSG (NOD-scid *IL2Rg^null^*) mice were obtained from the Jackson Laboratory (Bar Harbor, ME). All mouse strains except for NSG mice which were maintained by incross breeding were maintained on the C57BL/6 background under specific pathogen-free conditions at CARD, KU, and Comparative Medicine, National University of Singapore (NUS). All mouse experiments followed the institutional guidelines for the use of laboratory animals, and the experimental protocols for this study were approved by the Review Board for Animal Experiments of KU and NUS. Sequences of primers for genotyping are listed in Table S2.

**Serial collection of peripheral blood (PB) in mice**

To monitor changes in hematopoiesis, peripheral blood **(**PB) was serially collected by retroorbital bleeding under general anesthesia with indicated time intervals in Fig. 1B. Complete blood cell counts (CBC) were performed using an automated hemocytometer (MEK 6450, Nihon Kohden). PB was also subjected to an osmotic burst of red blood cells (RBCs) by lysis buffer (15 mM NH_4_Cl, 10 mM KHCO_3_, 0.1 mM Na_2_EDTA) on ice for 2-3 minutes, and subsequent flow cytometry analysis using FACS Canto II (BD Biosciences, San Jose, CA) to detect increase in GFP percentages in white blood cells (WBCs).

**Hematological analysis of mice at pre-leukemia and leukemia stages**

All hematological organs such as PB, bone marrow (BM), spleen, thymus and lymph nodes (LN) were harvested at asymptomatic pre-leukemic (1 month after tamoxifen injection) or premortal leukemia stages. Press-imprint-smear slides for spleen, thymus and LN, and blood smear were made for morphological analysis. Whole BM cells were isolated from femurs and tibias and filtered through a nylon filter (35 μm) to obtain single-cell suspension in phosphate-buffered saline (PBS) supplemented with 10% heat-inactivated fetal bovine serum (FBS). Other tissues were also similarly processed for single-cell suspension. 5-10 x10^4^ BM cells were cytospun on a slide using a cytocentrifuge (Cytospin 4, Thermo Fisher, MA). Morphology of malignant cells on cytospin or smear slides was examined after May-Grünwald-Giemsa staining.

Dissociated hematopoietic cells were suspended in PBS with 2% FBS at a concentration of 10^6^ cells/mL and stained on ice for 30 minutes with different antibody combinations (Table S3). The FITC channel was spared for GFP signal detection. After washing, the cells were suspended in 0.5 ml of PBS with Hoechst 33258 for dead cell discrimination. In order to identify immunophenotypic hematopoietic stem progenitor cells (HSPCs), lineage^+^ cells were negatively selected by staining with biotinylated lineage markers (Gr-1, Mac-1, CD4, CD8, B220, CD3 and TER119). In addition, cells were stained with Sca-1, c-Kit and CD34. An antibody against Flt3 was used to further fractionate c-Kit^+^Sca-1^+^Lin^-^ (KSL) population, while FcγR antibody was utilized to fractionate c-Kit^+^Sca-1^-^Lin^-^ (KL) population. A common lymphoid progenitor (CLP) population was identified using IL7Ra antibody. Stained single cells were subjected to multicolor flow cytometry analysis using FACS Canto II, or LSRII, or to sorting by FACSAria or FACSAria II (BD Biosciences, San Jose, CA) as previously described.^4^ Graphs and statistical analyses were conducted by FlowJo software version 10.4.2 (FlowJo, LLC, Ashland, OR).

**Bone marrow transplantation (BMT)**

BM cells from donor diseased mice (CD45.2/CD45.2) were collected in α-MEM media and then transplanted by tail vein injection into sublethally (2 Gy) irradiated NSG recipient mice (CD45.1/CD45.1) between 8 to 24 weeks of age. Chimerism of donor cells was assessed serially at various post-transplantation time points by analyzing the percentage of CD45.2^+^ and GFP^+^ cells in the PB by flow cytometric analysis. Diseased recipient mice were subjected to CBC, flow cytometry, and cytospin, like the primary diseased mice.

**Excision check of LSL cassette by PCR**

Genomic DNAs (gDNAs) were extracted from the BM or spleen using the phenol-chloroform method. PCR was performed on 10 ng of gDNA by GoTaq^®^ Master mix (Promega, Madison, WI) using primers listed in Table S2.

**Quantitative reverse transcription-PCR (qRT-PCR)**

RNAs from bulk or sorted hematopoietic cells were extracted using TRIZOL Reagent (Invitrogen, USA) and cDNAs were synthesized by EvoScript Reverse Transcriptase (Roche Diagnostics, Germany) according to the manufacturer's instructions. Real-time PCR was then performed using QuantStudio-3 Real-time PCR system (Applied Biosystems, Foster City, CA, USA) with an initial step of 95°C for 10 minutes followed by 40 cycles of 94°C for 15 seconds and 60°C for 1 minute. The specific primers for SYBR Green real-time PCR are listed in Table S2. All reactions were performed in duplicates or triplicates. Relative expression was calculated by comparative CT method using Glyceraldehyde-3-phosphate dehydrogenase (Gapdh) as an endogenous internal control.

**Western blot analysis**

Expression at protein level was determined by western blot analysis as previously described.^5^ Antibodies against RUNX1 (1:500, #4334S, Cell Signaling Technology, MA, USA), GFP (1:500, #598, MBL Life Science, Nagoya, Japan), and β-actin (1:10000, AC-15, ab6276, Abcam, Cambridge, UK) were used for immunoblotting.

**Cell lines and retroviral vector transduction**

Kasumi-1 and SKNO-1 cells are both human AML cell lines carrying t(8;21) and hence expressing the *RUNX1-ETO* fusion gene. These AML cell lines were cultured in RPMI-1640 medium with 20% or 10% fetal bovine serum (FBS). In addition, 10 ng/mL human granulocyte macrophage colony-stimulating factor (hGM-CSF) was supplemented for SKNO-1 cells. Mouse hematopoietic progenitor, EML cells, were cultured in IMDM medium with 20% FBS and 200 ng/mL mouse stem cell factor (mSCF). EML cells were transfected with the retroviral vector MIG [MSCV/Internal ribosomal entry site (IRES)/green fluorescent protein (GFP)] without any gene of interest (mock) or MIG with *RUNX-ETO9a*, as previously described.^6^

**Patient samples**

RUNX1-ETO expression at mRNA level was analyzed in BM mononuclear cells collected from AML with t(8;21) (n=8). Each patient gave informed consent to this study based on the tenets of the revised Helsinki protocol produced by the Institutional Committees for the Protection of Human Subjects and Analysis of the Human Genome. This study was approved by the institutional review board of Kumamoto University Hospital.

**Statistical analysis**

Data were analyzed using GraphPad Prism version 8.0. Differences evaluated by unpaired two-tailed student’s t-test. Kaplan-Meier analysis with Log-rank (Mantel-Cox) test was used for survival comparison. Multiple comparisons were calculated by one-way ANOVA test. p-values < 0.05 were considered statistically significant.

**SUPLEMENTARY REFERENCES**

1. Ishikawa E, Kosako H, Yasuda T, Ohmuraya M, Araki K, Kurosaki T, et al. Protein kinase D regulates positive selection of CD4+ thymocytes through phosphorylation of SHP-1. *Nat Commun*. 2016;7:12756.
2. Matsuo J, Kimura S, Yamamura A, Koh CP, Hossain MZ, Heng DL, et al. Identification of stem cells in the epithelium of the stomach corpus and antrum of mice. *Gastroenterology*. 2017;152(1):218-231.
3. Haigis KM, Kendall KR, Wang Y, Cheung A, Haigis MC, Clickman JN, et al. Differential effects of oncogenic K-Ras and N-Ras on proliferation, differentiation and tumor progression in the colon. *Nat Genet*. 2008;40(5):600-8.
4. Wang CQ, Motoda L, Satake M, Ito Y, Taniuchi I, Tergaonkar V, et al. Runx3 deficiency results in myeloproliferative disorder in aged mice. *Blood*. 2013;122(4):562-566.
5. Wang CQ, Krishnan V, Tay LS, Chin DWL, Koh CP, Chooi JY, et al. Disruption of Runx1 and Runx3 leads to bone marrow failure and leukemia predisposition due to transcriptional and DNA repair defects. Cell Rep 2014;8(3), 767-82.
6. Yamashita N, Osato M, Huang L, Yanagida M, Kogan SC, Iwasaki M, et al. Haploinsufficiency of Runx1/AML1 promotes myeloid features and leukaemogenesis in BXH2 mice. Br J Haematol 2005:131(4):495-507.

**Table S1. List of sgRNA Oligonucleotides.**

| CRISPR vector | Oligo sequence |
| --- | --- |
| pX335-Rosa26-3 | 5’- caccgTGGGCGGGAGTCTTCTGGGC-3’ |
|  | 5’- aaacGCCCAGAAGACTCCCGCCCAc-3’ |
| pX335-Rosa26-4 | 5’- caccgACTGGAGTTGCAGATCACG -3’ |
|  | 5’- aaacCGTGATCTGCAACTCCAGTc -3’ |

**Table S2. List of primers.**

| **Mouse strain** | **Purpose** | **Primer name** | **Sequence** |
| --- | --- | --- | --- |
| eR1-CreER^T2^ Tg | Genotyping | eR1 F | 5’- CACTGATAACGTGGGCAGCTT -3’ |
|  |  | mhsp68 R | 5’- GTGTCCGGTGACGTGATCCTC -3’ |
| RUNX1-ETO | Genotyping | RE F | 5’- TCGGCTGAGCTGAGAAATGCTA -3’ |
|  |  | RE R | 5’- TGAAGCCATTGGGTGGTGA -3’ |
|  | Excision | RE-Ex F1 | 5’- GAGATCATAATCAGCCATACCAC -3’ |
|  |  | RE-Ex F2 | 5’- GGTTGAGGACAAACTCTTCG -3’ |
|  |  | RE-Ex R | 5’- CACGGAGCAGAGGAAGTTG -3’ |
|  | qRT-PCR | RE F | 5’- TACCACAGAGCCATCAAAATCAC -3’ |
|  |  | RE R | 5’- ATTGCGTCTTCACATCCACAG -3’ |
| Nras^G12D^ | Genotyping | Nras F2 | 5’- AGACGCGGAGACTTGGCGAGC -3’ |
|  |  | Nras R1 | 5’- GCTGGATCGTCAAGGCGCTTTTCC -3’ |
|  |  | Nras SD5 | 5’-AGCTAGCCACCATGGCTTGAGTAAGTC TGCA -3’ |

**Table S3. Antibodies for flow cytometry.**

| Antigen | Clone | Conjugated fluorochrome | Manufacture |
| --- | --- | --- | --- |
| CD117 (c-Kit) | 2B8 | BV-421 | BD Biosciences |
| CD117 (c-Kit) | 2B8 | APC | BioLegend |
| CD11b | M1/70 | Biotin | BD Biosciences |
| CD11b (Mac-1) | M1/70 | PerCP-Cy 5.5 | BD Biosciences |
| CD135 | A2F10 | PE | BD Biosciences |
| CD16/32 | 93 | PE | BioLegend |
| CD19 | 1D3/CD19 | PE | BioLegend |
| CD25 | PC61 | APC | BD Biosciences |
| CD34 | RAM 34 | AF-647 | BD Biosciences |
| CD3e | 145-2C11 | PerCP-Cy5.5 | BioLegend |
| CD3e | 145.2C 11 | Biotin | BD Biosciences |
| CD4 | RM4-5 | APC | BioLegend |
| CD4 | GK 1.5 | Biotin | BD Biosciences |
| CD41 | MWReg30 | APC-Cy7 | BioLegend |
| CD44 | IM7 | APC-Cy7 | BioLegend |
| CD45R/B220 | RA3-6B2 | PE-Cy7 | BD Biosciences |
| CD45R/B220 | RA3-6B2 | Biotin | BD Biosciences |
| CD45R/B220 | RA3-6B2 | BV510 | BD Biosciences |
| CD49b | DX5 | PE-Cy7 | BioLegend |
| CD61 | 2C9.G2(HMβ3-1) | PE-Cy7 | BioLegend |
| CD71 | C2 | PerCP-Cy 5.5 | BD Biosciences |
| CD8a | 53-6.7 | APC-Cy7 | BD Biosciences |
| CD8a | 53-6.7 | Biotin | BD Biosciences |
| Fas (CD95) | SA367H8 | PerCP-Cy5.5 | BioLegend |
| Gr-1 | RB6-8C5 | PE | BD Biosciences |
| Gr-1 | RB6-8C5 | PerCP-Cy5.5 | ebioscience |
| IL7R CD127 | A7R34 | PE | ebioscience |
| Ly6G-Ly.6C | R86-8C5 | Biotin | BD Biosciences |
| Sca-1 | D7 | PE-Cy7 | BD Biosciences |
| Streptavidin |  | APC-Cy7 | BioLegend |
| TCRαβ | H57-597 | PE | BioLegend |
| TCRγδ | GL3 | PE-Cy7 | BioLegend |
| TER-119 | TER119 | PE | BD Biosciences |
| TER-119 | TER119 | Biotin | BD Biosciences |
| CD45.1 | A20 | PE | BioLegend |
| CD45.2 | 104 | APC-Cy7 | BioLegend |

**Table S4. Classification criteria for disease subtypes.**

| Disease subtype | % GFP* | Immunophenotype** | |
| --- | --- | --- | --- |
|  |  | **Gr-1** | **B220** |
| AML M1 | ≥10 | ˂ 30 | ˂ 30 |
| AML M2a | ≥10 | ≥30 | ˂ 30 |
| AML M2b | ≥ 10 | ≥30 | ≥30 |
| MPD a | ˂ 10 | ≥30 | ˂ 30 |
| MPD b | ˂ 10 | ≥30 | ≥30 |

* in any single hematopoietic tissue (PB, BM, spleen or thymus). For the objective classification of mouse disease subtypes, Euclidian distance analysis using %GFP and immunophenotype data was conducted. By setting 10% of GFP^+^ cells as a cut-off value, hierarchical clustering analysis demonstrated that most MPD cases were segregated from AML cases. In addition, within the AML cases, individual leukemia subtypes were also nicely clustered together. Therefore, the 10% GFP appears to serve as an objective cut-off to some extent.

** % in GFP^+^ population for AML, while % in total BM for MPD

**LEGENDS FOR SUPLEMENTARY FIGURES**

**Figure S1. Rosa26-RUNX1-ETO has normal hematopoiesis with age dependent alterations of differentiation.** (A and B) Cre-mediated excision efficiency of the LoxP-Stop-LoxP (LSL) cassette in the bone marrow (BM) cells from eR1CreER^T2^;Rosa26-LSL-RUNX1-ETO-IRES-EGFP mouse model, after 24 hours (hr) of 0.05 mg/g tamoxifen (TMX) injection. Representative flow cytometry profiles (A) and averages of the frequency of GFP^+^ cells in c-Kit^+^Sca-1^-^Lin^-^ (KL) and c-Kit^+^Sca-1^+^Lin^-^ (KSL) populations at postnatal day 3 (P3) and 4-week (4w) cohorts (B) are shown. (P3, n=2; 4w, n=2) (C-F) Hematological status at asymptomatic pre-leukemic stage, examined by flow cytometry analysis. (C) Mean frequencies of GFP^+^ cells in the BM, spleen and thymus after 4 weeks of TMX injection in three indicated age cohorts (P3, n= 4; 2w, n=4; 4w, n=3). (D) Heatmaps for mean frequencies of indicated antigen^+^ cells within GFP^+^ population in the BM (left panel) and total BM cells (right panel). White or black asterisk(s) represents significant increase or decrease respectively, against the corresponding WT population [*P ≤ 0.05, two-way ANOVA with subsequent Bonferroni test]. (E) Bar graphs depicting differences in indicated HSPC fractions between wild type versus RUNX1-ETO^+^ pre-leukemic cells from P3 cohort, and the contribution of GFP^+^ and GFP^-^ cells to individual HSPC compartments. (F) Heatmaps for mean frequencies of indicated population of hematopoietic stem progenitor cells (HSPCs) within GFP^+^ population in the BM (left panel) and total BM cells (right panel). Asterisk(s): see legend for (D). (G) Spider plots depicting time course kinetics of white blood cells (WBC) (upper panels) and percentages of GFP^+^ cells (lower panels) in peripheral blood (PB) in individual diseased mice. Data from acute myeloid leukemia (AML) and myeloproliferative disorder (MPD) are shown in the left and right panels separately. Line colors correspond to disease subtypes or wild type (WT). Abbreviations: see legend for Fig. 1.

**Figure S2. Immunophenotype of RUNX1-ETO induced malignant cells.** (A-D) Heatmaps for percentages of indicated antigen^+^ cells in individual diseased mouse, within GFP^+^ population in the BM (A) and total BM cells (B). Similarly, those in the spleen are shown in (C) and (D). Diseased mice are grouped according to their subtypes shown at the top of individual panels. For comparison, percentages of indicated antigen^+^ cells in wild type mice are shown in B and D. (E-G) Remaining AMP in the final diseases status. (E) Representative flow cytometry contour plots of AMP (red gated) in KL compartment of diseased mice from indicated subtypes, and histograms for %GFP in the corresponding AMP are shown. Mean frequencies of AMP in KL (F) and in total BM (G) in various disease subtypes are shown (sample size: WT, 5; M1, 4; M2a, 6; M2b, 4; MPD a, 3; MPD b, 1). Abbreviations: AML, acute myeloid leukemia, MPD, myeloproliferative disorder; others, see legend for Fig. 1.

**Figure S3. RUNX1-ETO induces MPD in a non-cell autonomous manner.** (A) Representative May-Grunwald-Giemsa staining of cytospun bone marrow cells from indicated disease subtypes. (B) Correlation between the extent of LSL cassette excision and %GFP in MPD cases. Polymerase chain reaction (PCR), conducted for genomic DNAs extracted from BM cells of MPD cases, amplified DNA fragments of 527 bp and 308 bp from non-excised and excised alleles, respectively. Wild type (WT), non-TMX injected RUNX1-ETO mice, and AML mice of indicated %GFP (3.1, 10.7 and 40%) were used as negative and positive controls (lanes 1-5). Results of 6 MPD cases are shown in lanes 6-11. Lane M denotes molecular weight size marker. Intensities of excised bands demonstrate a good correlation with %GFP in MPD cases, with a minimum detection threshold of around 3% in GFP. (C) Correlation between RUNX1-ETO mRNA expression and %GFP in MPD cases. Expression levels of RUNX1-ETO mRNA were examined by quantitative reverse transcription-PCR (qRT-PCR). Negative and positive controls are identical to those in C. Results of qRT-PCR for 6 MPD cases show a good correlation with %GFP stated below of the graph. (D) Comparison of RUNX1-ETO mRNA expression between immature and mature cells. qRT-PCR assay was conducted for c-Kit^+^Gr-1^-^B220^-^ blast cells, c-Kit^-^Gr-1^+^B220^-^ granulocytes, and c-Kit^-^Gr-1^-^B220^+^ B cells in GFP^+^ or GFP^-^ fractions, sorted from diseased mice. Asterisk(s) represents significant differences [*P ≤ 0.05, **P ≤ 0.01, unpaired two-tailed student’s t-test]. (E) Kaplan-Meier survival curves of individual disease subtypes. Asterisk(s) represents significant differences (*P ≤ 0.05, **P ≤ 0.01, ***P ≤ 0.001, Log-rank test). (F) Schematic summary of two distinct potential mechanisms for the development of RUNX1-ETO mediated malignancies in this mouse model. In most cases, RUNX-ETO induction at the early childhood stage results in massive expansion of AMP with modest changes in GMP, granulocytes and B lymphocytes, at asymptomatic pre-leukemic state, and eventually causes acute myeloid leukemia (AML) in a relatively short period. In contrast, RUNX-ETO positive cells induced at late childhood stage or older remain as a small subset in the particular mice and may lead to MPD with a long latency in a non-cell autonomous manner.

**Figure S4. RUNX1-ETO GFP^+^, but not GFP^-^, leukemia cells induces leukemia when transplanted.** (A) Schematic summary for workflow and result of transplantation assay. Whole bone marrow cells (WBM), sorted GFP^+^ cells, or GFP^-^ cells collected from the primary diseased eR1-CreERT2; RUNX1-ETO (CD45.2) mice were transplanted into sublethally irradiated NSG (CD45.1) mice. Disease subtypes in primary (donor) mice and secondary (recipient) mice are shown in respective sections. One particular row represents an experimental pair of a single donor and its respective recipients. For example, in the first row, leukemia cells form one AML M2a caused M1 and M2a leukemia subtypes in two distinct recipient mice. Letter colors for disease subtypes, namely black, blue, green and red, represent experimental categories based on donor cells, such as WBM (AML), WBM (MPD), GFP^+^ and GFP^-^ fractions, respectively. Representative flow cytometry profiles for disease or non-disease mice are shown on the right. Top and middle profiles demonstrate the profile of AML M1 developed in one of the mice transplanted with WBM (AML) and AML M2b arose in one of the mice transplanted with GFP^+^ cells, respectively, whereas bottom profiles exhibit no disease development despite successful transplantation of CD45.2 donor cells in the peripheral blood (PB) in the CD45.1 NSG recipient mouse. (B) Behaviors of %GFP in the PB (left) and %GFP in donor cells (right) in the recipient mice (2°) as compared to those in the donor diseased mice (1°). Line colors: see legends for (A) on letter colors. (C) Kaplan-Meier survival curves of the reciepient NSG mice. WBM (AML), n= 4; WBM (MPD), n=4; GFP^+^, n= 3; GFP^-^, n=5. (D) Behaviors of indicated parameters in the recipient NSG mice (2°). Mean values at initial and latest time points are shown.

**Figure S5. Nras^G12D^ mutation accelerates RUNX1-ETO mediated leukemogenesis in mice like human patients.** Double mutant mice (Nras;RUNX1-ETO) reveals much faster increase in white blood cell (WBC) counts (A) and %GFP (B), shorter latency (C), and complete penetrance (D), as compared to corresponding single mutant RUNX1-ETO mice of 4-week cohort. Sample size: Nras;RUNX1-ETO, 6; RUNX1-ETO, 9. Asterisk(s) represents significant differences [*P ≤ 0.05, **P ≤ 0.01, ***P ≤ 0.001, unpaired two-tailed student’s t-test (A and B), Log-rank (Mantel-Cox) test (C)] (D) Stacked bar graph displaying frequency and spectrum of indicated hematological disease subtypes in Nras^G12D^;RUNX1-ETO and corresponding RUNX1-ETO mice.
